# Potential self-medication by brown titi monkeys, *Plecturocebus brunneus*, in an urban fragment forest in the Brazilian Amazon

**DOI:** 10.5194/pb-7-35-2020

**Published:** 2020-12-15

**Authors:** Brenda Letícia Pereira Oliveira, João Pedro Souza-Alves, Marcela Alvares Oliveira

**Affiliations:** 1 Curso de Ciências Biológicas, Centro Universitário Aparício Carvalho, Porto Velho, Rondônia,76811-678, Brazil; 2 Programa de Pós-Graduação em Biologia Animal, Universidade Federal de Pernambuco, Recife, 50670-901, Brazil; 3 Programa de Pós-Graduação em Biodiversidade e Biotecnologia da Amazônia Legal, Universidade Federal de Rondônia Rondônia, Porto Velho, 76812-245, Brazil; 4 Laboratório de Ecologia, Comportamento e Conservação (LECC), Departamento de Zoologia, Universidade Federal de Pernambuco, Recife, 50670-901, Brazil

## Abstract

In this study, we report fur-rubbing behavior of brown titi monkeys, *Plecturocebus brunneus, *using chewed leaves from (Fabaceae) and *Piper tuberculatum* (Piperaceae). These reports were obtained during systematic monitoring of titi monkeys from May until December 2019 (218 h) in an urban fragment forest in the Brazilian Amazon. Both plant species contain chemical substances in their leaves that potentially repel ectoparasites. The genus *Piper* is known for its repelling action due to the presence of amides, alkaloids and benzoic acid. The presence of dogs, cats and human settlements may contribute to an increase of ectoparasites, making a potential self-medication function of fur rubbing in this primate species plausible.

## 1 Introduction

The fur-rubbing or self-anointing behavior from different plant parts and insects is relatively well documented in wild and captive Neotropical primates. Overall, this behavior may be associated with several functions such as repelling or killing of ectoparasites and microbial pathogens (self-medication), and scent marking (olfactory communication) (Huffman et al., 2013; Martinez et al., 2019). The chemical substances emerging from plants and arthropods, after being chewed and crushed, act as therapeutic medicine for the species (Huffman, 2011) due to the presence of metabolic compounds with inhibitory action (therapeutic action) on parasites (Peckre et al., 2018).

Several plant genera are frequently used by primates in self-medication events. Capuchins (*Cebus capucinus*), for example, use *Citrus*, *Sloanea*, *Clematis* and *Piper* plants (Baker, 1996), captive and wild titi monkeys *Piper* and *Bauhinia* (Huashuayo-Llamocca and Heymann, 2017; Souza-Alves et al., 2018, respectively), and spider monkeys (*Ateles geoffroyi*) *Citrus* and *Zanthoxylum* (Campbel, 2000). Arthropods are also used often, as for example millipedes can secrete caustic benzoquinones that work quickly on the fur and body of primates and are repellent to insects (Valderrama et al., 2000). Such actions tend to increase in frequency during the rainy period when the risks of insect bites and infections are higher (Huffman, 1997).

Titi monkeys are small-bodied primates living in family groups consisting of a breeding pair, one juvenile and one offspring (Bicca-Marques and Heymann, 2013). They devote most of their activity time to resting and feed mainly on fleshy fruits (Bicca-Marques and Heymann, 2013). Fur-rubbing behavior in titi monkeys has been documented and interpreted both as self-medication (Carrillo-Bilbao et al., 2005; Huashuayo-Llamocca and Heymann, 2017) and olfactory communication (Souza-Alves et al., 2018). The brown titi monkey, *Plecturocebus brunneus*, is distributed across the tropical forest from Bolivia, Brazil and Peru (Veiga et al., 2008). In Brazil, this species is endemic in the state of Rondônia, inhabiting continuous areas of Amazonian forests as well as urban forest fragments (Ferrari et al., 2000), where it can be found in small fragments (<1 km${}^{2})$ (Medeiro et al., 2019). Most research on these Brazilian populations of *P. brunneus* focused on their geographical distribution and general behavioral activities (Ferrari et al., 2000; Souza-Alves et al., 2019). Consequently, reporting rare behaviors such as fur rubbing increases our understanding of the socioecology of this species. Here, we report fur-rubbing behavior in one group of *P. brunneus* in an urban forest fragment in the South-Occidental Amazon.

## 2 Methods

Our reports were performed during systematic monitoring conducted in an urban fragment of the Amazon in the municipality of Porto Velho (8${}^{\circ }$44${}^{\prime }$33 36${}^{\prime \prime }$ 5 S, 63${}^{\circ }$52${}^{\prime }$18 50${}^{\prime \prime }$ 0), northern Brazil. The urban forest is categorized as a protected area according to Brazilian Forest Law (decree number 12.651/2012). Although the area is legally protected it suffers negative impact and size reduction due to anthropogenic disturbances. Adjoining the area is a car wash and houses without a sewage treatment network, acting as constant sources of solid waste and water, a common condition in different urban fragments of the municipality (Moreira and Oliveira, 2019). Currently, the forest fragment is comprised of ca. 2000 km${}^{2}$ composed of secondary vegetation with a high richness of exotic plant species such as *Musa paradisiaca* (banana) and *Persea americana* (avocado). The mean annual rainfall varies between 2000 and 2300 mm, with high temperatures from 24 to 27 ${}^{\circ }$C across the year. The dry season is short, encompassing 2 and 3 months (July–August), reaching maximum temperatures up to 37 ${}^{\circ }$ C (Mendonça and Danni-Oliveira, 2014).

**Figure 1:**
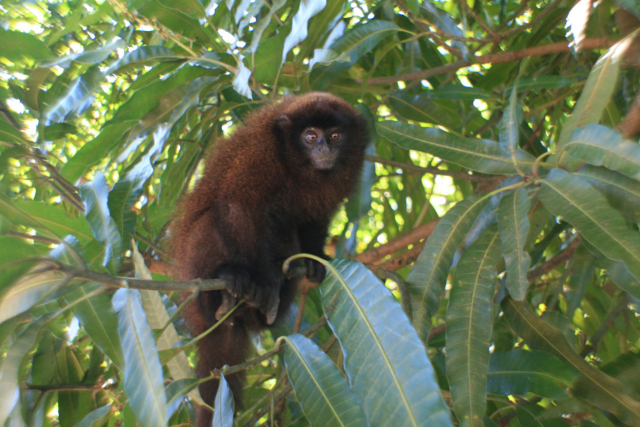
Juvenile member of brown titi monkey (*Plecturocebus brunneus*) study group in the urban fragment forest. Photo: Rallison Viamonte.

The titi monkey group was monitored from May to December 2019 on 3 d per month between 08:00 and 15:00 AMT (Amazon time, UTC-4) yielding a total of 218 observation hours across 35 d. During this period the study group was composed of three individuals: a breeding pair and a juvenile of unidentified sex (Fig. 1). Fur-rubbing behaviors were recorded ad libitum (Altmann, 1974). When possible, the plant species and specific parts used were identified.

## 3 Results

Fur-rubbing behavior was reported on a total of 27 occasions. On 12 occasions (8 times by the adult male, 4 times by the adult female) titi monkeys used *Senna obtusifolia* (Fabaceae) leaves, and on the remaining 15 occasions (10 times by the adult male, 5 times by the adult female) they used *Piper tuberculatum* (Piperaceae) leaves. Using *S. obstusifolia* leaves, individuals picked the leaves from the tree, chewed them and subsequently rubbed the chewed leaves on their chest. This behavior was usually seen when the individuals were alone on a tree branch and during the morning two to three times. At no time did titi monkeys rub their bodies on tree branches or against the body of group members after chewing the leaves.

During fur-rubbing behavior using *P. tuberculatum* leaves, all members of the study group were present. Individuals removed leaves, chewed them for a short period (2–5 s) and rubbed the chewed leaves on both hands. It was not observed whether the individuals rubbed their hands subsequently on tree branches or other individuals. All these events were observed in the morning between 08:00 and 10:00 AMT (Amazon time, UTC-4).

## 4 Discussion

From anecdotal reports, we described the fur-rubbing behavior in a poorly studied species of titi monkey, *Plecturocebus brunneus*, in an urban Amazonian fragment forest in Brazil. Owing to the chemical characteristics of the plant species, it seems likely that this behavior is a form of self-medication. *Senna obtusifolia* is an invasive species in Brazil with possible origin in the Caribbean and the northern region of South America (Randell, 1995). It has different allelopathic properties (e.g., Abdullahi et al., 2017), and among humans, it is used in traditional medicine as treatment for gingivitis, urinary tract infections, diarrhea, fever and cough (Doughari et al., 2008; Mahwasanel et al., 2013). Toxicity of the leaf extract of *Senna* presents an antimicrobial action with the presence of saponins, tannins, alkaloids and flavonoids (Doughari et al., 2008). Piperaceae is considered a pantropical family, with species being found from Mexico to southeastern Argentina (Figueiredo and Sazima, 2000). Medical use includes several treatments such as recovery from childbirth, toothache, stomach pain, colds and symptoms of erysipelas – a bacterial infection in the upper layer of the skin (Martínez-Bautista et al., 2019). *Piper tuberculatum* has proven to act in laboratory tests as an anti-drug (Silva Lima et al., 2014), anti-tumor (Bezerra et al., 2015), anti-leishmania (Ferreira et al., 2010) and as an insecticide (Bazán-Calderón et al., 2011). This genus presents various biological compounds such as amides, alkaloids and benzoic acid (Parmar et al., 1997). *P. tuberculatum* leaves are composed of 4,5‐dihydropiperlonguminine, piperlongumine, 4,5‐dihydropiperine and piperine (Scott et al., 2002). These chemical substances demonstrate a strong insecticide activity (Bazán-Calderón et al., 2011).

Although leaves of *Senna obtusifolia* present chemical compounds with the ability to act on different diseases, their role in self-medication of *P. brunneus* remains unclear. In contrast, behavior similar to the one reported here has been observed previously in titi monkeys using different *Piper* species (Huashuayo-Llamocca and Heymann, 2017; Martínez et al., 2019). Due to the insecticidal properties of different species of the genus *Piper* and the characteristics of the method of use, both studies concluded this behavior to be related to self-medication in order to repel insects. As such, it seems reasonably likely that the events recorded for *P. brunneus* in this study represent self-medication behavior.

Further evidence is needed to determine the direct stimulus for fur rubbing at this site. Additionally, the characteristics of the site, such as the presence of housing (including humans, dogs and cats) and the constant disposal of water can favor the reproduction of ectoparasites (Solórzano-Garcia and Pérez-Ponce de Léon, 2018), which may lead to individuals of *P. brunneus* to performing fur-rubbing self-medication behavior. Martínez et al. (2019) point out that the long and dense fur of the titi monkeys acts as a barrier to the action of insects, but the hands, neck and chest would be devoid of this type of protection. As titi monkeys in this study used the products of chewing on the hands and breast region and both plants have different allelopathic functions, this behavior reinforces the possibility of using these plants as a repellent from parasites or relief from their irritative effect.

Anecdotal evidence often points to characteristics that can be later confirmed through more rigorous scientific analysis (Browning, 2017). In this context, it is plausible to speculate that the use of the leaves of *S. obtusifolia* and *P. tuberculatum* used for the *P. brunneus* is associated with self-medication behavior (Huffman, 1997).

## Data Availability

There are no further data apart from the observations reported in the paper.
